# Using Multivariate Regression Model with Least Absolute Shrinkage and Selection Operator (LASSO) to Predict the Incidence of Xerostomia after Intensity-Modulated Radiotherapy for Head and Neck Cancer

**DOI:** 10.1371/journal.pone.0089700

**Published:** 2014-02-28

**Authors:** Tsair-Fwu Lee, Pei-Ju Chao, Hui-Min Ting, Liyun Chang, Yu-Jie Huang, Jia-Ming Wu, Hung-Yu Wang, Mong-Fong Horng, Chun-Ming Chang, Jen-Hong Lan, Ya-Yu Huang, Fu-Min Fang, Stephen Wan Leung

**Affiliations:** 1 Medical Physics and Informatics Laboratory of Electronics Engineering, National Kaohsiung University of Applied Sciences, Kaohsiung, Taiwan, ROC; 2 Department of Radiation Oncology, Kaohsiung Chang Gung Memorial Hospital and Chang Gung University College of Medicine, Kaohsiung, Taiwan, ROC; 3 Department of Electrical Engineering, National Kaohsiung University of Applied Sciences, Kaohsiung, Taiwan, ROC; 4 Department of Medical Imaging and Radiological Sciences, I-Shou University, Kaohsiung, Taiwan, ROC; 5 Department of Radiation Oncology, E-Da Hospital, Kaohsiung, Taiwan, ROC; 6 Department of Electronics Engineering, National Kaohsiung University of Applied Sciences, Kaohsiung, Taiwan, ROC; 7 Department of Radiation Oncology, Kaohsiung Yuan's General Hospital, Kaohsiung, Taiwan, ROC; Dresden University of Technology, Germany

## Abstract

**Purpose:**

The aim of this study was to develop a multivariate logistic regression model with least absolute shrinkage and selection operator (LASSO) to make valid predictions about the incidence of moderate-to-severe patient-rated xerostomia among head and neck cancer (HNC) patients treated with IMRT.

**Methods and Materials:**

Quality of life questionnaire datasets from 206 patients with HNC were analyzed. The European Organization for Research and Treatment of Cancer QLQ-H&N35 and QLQ-C30 questionnaires were used as the endpoint evaluation. The primary endpoint (grade 3^+^ xerostomia) was defined as moderate-to-severe xerostomia at 3 (XER_3m_) and 12 months (XER_12m_) after the completion of IMRT. Normal tissue complication probability (NTCP) models were developed. The optimal and suboptimal numbers of prognostic factors for a multivariate logistic regression model were determined using the LASSO with bootstrapping technique. Statistical analysis was performed using the scaled Brier score, Nagelkerke R^2^, chi-squared test, Omnibus, Hosmer-Lemeshow test, and the AUC.

**Results:**

Eight prognostic factors were selected by LASSO for the 3-month time point: Dmean-c, Dmean-i, age, financial status, T stage, AJCC stage, smoking, and education. Nine prognostic factors were selected for the 12-month time point: Dmean-i, education, Dmean-c, smoking, T stage, baseline xerostomia, alcohol abuse, family history, and node classification. In the selection of the suboptimal number of prognostic factors by LASSO, three suboptimal prognostic factors were fine-tuned by Hosmer-Lemeshow test and AUC, i.e., Dmean-c, Dmean-i, and age for the 3-month time point. Five suboptimal prognostic factors were also selected for the 12-month time point, i.e., Dmean-i, education, Dmean-c, smoking, and T stage. The overall performance for both time points of the NTCP model in terms of scaled Brier score, Omnibus, and Nagelkerke R^2^ was satisfactory and corresponded well with the expected values.

**Conclusions:**

Multivariate NTCP models with LASSO can be used to predict patient-rated xerostomia after IMRT.

## Introduction

Head and neck cancers (HNC) are a leading cause of cancer mortality in Taiwan. Radiotherapy (RT) plays an important role in the treatment of HNC. However, xerostomia is a common complication in patients with HNC after radiotherapy. According to the Late Effects of Normal Tissues – Subjective, Objective, Management, Analytic (LENT-SOMA) criteria, severe xerostomia is defined as long-term salivary function <25% of the pre-RT baseline value [1], [2]. Based on the quantitative analysis of normal tissue effects in the clinic (QUANTEC) guideline to limit the incidence of severe xerostomia to below 20%, at least one parotid gland should receive a mean dose of ≤20 Gy, or both parotid glands should receive a mean dose of ≤25 Gy [1], [2]. The patient-rated quality of life (QoL) questionnaire (QLQ-C30) and the xerostomia-specific QoL questionnaire (QLQ-H&N35) have been shown to allow a reliable assessment of the relationships between QoL and salivary function and xerostomia in patients receiving radiotherapy [3]–[6]. The normal tissue complication probability (NTCP) model was developed using either a univariate or multivariate logistic regression model to predict the incidence of xerostomia. However, the development of xerostomia as reported by patients most likely depends on a variety of prognostic factors [3]–[5], [7], [8]. Some variables such as clinical and treatment-related factors that may have important effects on the risk of radiation-induced complications need to be taken into consideration. Therefore, one goal of this study was to develop predictive models for patient-rated xerostomia, taking into account dose distributions in parotid glands as well as other potential clinical and treatment-related prognostic factors.

Developing a multivariate logistic regression model requires knowing the optimal number of prognostic factors to include. Many investigators used the log likelihood (LL), average likelihood, Akaike information criterion (AIC), and Bayesian information criterion (BIC) to deal with this problem [3]. Xu et al. [9]–[11] introduced the least absolute shrinkage and selection operator (LASSO) and Bayesian model averaging (BMA) to build NTCP models of xerostomia after three-dimensional conformal radiation therapy (3D-CRT) for HNC. However, the BMA needs a priori information to build a fair predictive model. The LASSO is based on shrinkage estimation and has been widely used in the statistical field [12]–[16]. The advantages of LASSO include: 1) a smaller mean squared error (MSE) than conventional methods; 2) it handles the multicollinearity problem; 3) overall variable selection; and 4) coefficients shrink [9]–[11]. Being easy to implement is another of the merits that attract users. Xu et al. recommended the LASSO method for multivariate logistic regression NTCP modeling [9].

Beetz et al. showed that 3D-CRT-based models for patient-rated xerostomia among HNC patients treated with primary RT turned out to be less valid for patients treated with intensity-modulated radiotherapy (IMRT), and that the 3D-CRT NTCP models cannot be used for IMRT cohorts [5]. The main message was that models developed in a population treated with a specific technique cannot be generalized and extrapolated to a population treated with another technique without external validation. Therefore, the purpose of this study was to develop a multivariate logistic regression model with LASSO to make valid predictions about the incidence of patient-rated xerostomia among HNC patients treated with IMRT at 3 and 12 months after treatment.

## Materials and Methods

### Study population

We aimed to develop a multivariate logistic regression NTCP model with LASSO to make valid predictions about the risk of moderate-to-severe patient-rated xerostomia using QoL datasets. In total, 206 patients with HNC were enrolled. All participants were treated with IMRT at the Kaohsiung Chang Gung Memorial Hospital between May 2007 and October 2010. The characteristics of the 206 patients with HNC are presented in Table 1. QoL questionnaires completed by patients prior to treatment and at 3 and 12 months after treatment were analyzed and used for multivariate logistic regression NTCP model response fitting. This study was approved by the Chang Gung medical foundation institutional review board (99-1420B, 96-1231B) and all participants gave written informed consent.

**Table 1:** Characteristics of patients with head and neck cancer treated by IMRT.

	Value—x (%)
	HNC (n = 206)
**Age (y)**	
Mean	52
Range	26–89
**Gender (** ***n*** **)**	
Male	178 (86.4%)
Female	28 (13.6%)
**Tumor site**	
Larynx	14 (6.8%)
Hypopharynx	15 (7.3%)
Oropharynx	37 (18%)
Oral cavity	53 (25.7%)
Nasopharyngeal carcinoma	84 (40.8%)
Other	3 (1.4%)
**AJCC stage**	
I	11 (5.3%)
II	35 (17%)
III	43 (20.9%)
IV	117 (56.8%)
**Total dose**	
40–60	11(5.3%)
60–65	48(23.3%)
65–70	79(38.3%)
70–75	58(28.2%)
75–80	10(4.9%)
**QoL measurement at 3 months after IMRT**	206
Grade 3^+^ xerostomia	87 (42.2%)
No grade 3^+^ xerostomia	98 (47.6%)
With grade 3^+^ xerostomia at baseline	21 (10.2%)
**QoL measurement at 12 months after IMRT**	128
Grade 3^+^ xerostomia	43 (33.6%)
No grade 3^+^ xerostomia	74 (57.8%)
With grade 3^+^ xerostomia at baseline	11 (8.6%)
**Chemotherapy**	
Yes	166(80.6%)
No	40(19.4%)

*Abbreviation:* AJCC: American Joint Committee on Cancer; QoL: quality of life; IMRT: intensity-modulated radiotherapy;

Grade 3^+^ xerostomia was defined as moderate (66) to severe (100) xerostomia 3 and 12 months after the completion of RT, and those patients with moderate to severe xerostomia at baseline were excluded from the analysis.

### IMRT techniques

Each patient was immobilized using a commercially available thermoplastic mask and/or an individually customized bite block. Computed tomographic images (2.5-mm slice thickness, 512×512 pixels/slice) were acquired from the top of the vertex to the level of the carina. Both parotid glands were delineated by a radiation oncologist. Dose distributions were calculated and dose–volume histograms (DVHs) were generated separately for each parotid gland, enabling separate analysis. Two IMRT techniques were used: simultaneous integrated boost (SIB) and sequential mode (SQM). The prescribed total dose ranged from 54.0 to 77.4 Gy (median, 70.0 Gy).

IMRT was delivered by the computer-controlled auto-sequencing segment or the dynamic multileaf collimator of a linear accelerator [Varian Clinac 21 EX (Varian Medical Systems, Palo Alto, CA) or Elekta Precise (Elekta, Crawley, UK)] as described previously [17], aiming to spare the parotid glands (predominantly contralateral side) while treating the primary targets and lymph nodes at risk. The prescribed doses were 54.0–77.4 Gy (median, 70.0 Gy) to the macroscopic tumor planning target volume (PTV1), 48.0–72.0 Gy (median, 61.2 Gy) to the resected tumor bed planning target volume (PTV2), and 41.4 Gy to the subclinical disease planning target volume (PTV3), at 1.6–2.12 Gy per fraction for SIB and 1.8–2.0 Gy per fraction for SQM and five fractions per week.

According to the Radiation Therapy Oncology Group 0225 and 0615 trials, the planning objectives for PTVs were a minimum dose >95%, and no more than 5% of any PTV1 received ≥110% of the prescribed dose. The structural constraints for the parotid gland were a mean dose ≤26 Gy or V_30Gy_≤50%; a mean dose ≤40 Gy was used in the oral cavity excluding the PTV. Mean DVH values were calculated for the parotid glands for every patient. All data were based on the DVHs obtained using Pinnacle^3^® (Philips, Fitchburg, WI) with a bin size resolution of 0.01 Gy. The resolution of dose calculation was 2.5 mm for all IMRT plans.

Details about the prescribed dose and fractions for the SIB and SQM techniques can be found in previous studies [18], [19].

### QoL evaluation

A prospective survey of QoL using the European Organization for Research and Treatment of Cancer (EORTC) C30 and H&N35 QoL questionnaires (QLQ-C30 and QLQ-H&N35) was performed on 206 survivors of HNC. The patients were asked to complete the questionnaire prior to treatment and 3 months, 6 months, 1 year, and 2 years after IMRT. For the purposes of this analysis, the 3-month (*n* = 206) and 12-month (*n* = 128) follow-up time points were used. Chinese versions of the EORTC QLQ-C30 and QLQ-H&N35 questionnaires were obtained from the Quality of Life Unit, EORTC Data Center, Brussels, Belgium [20]. For each item on the EORTC QLQ-C30 and QLQ-H&N35 questionnaires, the following four-point Likert scale was used: none (0), a little (33), quite a lot (66), and a lot (100). All QoL scores are given in the text. A high score on the functional or global QoL scale represents a relatively high/healthy level of functioning or global QoL, whereas a high score on the symptom scale represents the presence of a symptom or problem. The EORTC QLQ-H&N35 questionnaire was used to evaluate xerostomia (i.e., the analytical endpoint). Grade 3^+^ xerostomia was defined as moderate (66) to severe (100) xerostomia 3 and 12 months after the completion of RT; this corresponds to the two highest scores on the four-point Likert scale. As we were primarily interested in grade 3^+^ xerostomia induced by RT itself, patients with moderate–to-severe xerostomia at baseline were excluded from further analysis [3]–[5], [21]. The primary endpoint (grade 3^+^ xerostomia) was defined as moderate-to-severe xerostomia at 3 (XER_3m_) and 12 months (XER_12m_) after the completion of IMRT, and excluded patients with grade 3^+^ xerostomia at baseline.

### Statistical analysis

NTCP models for moderate-to-severe patient-rated xerostomia were developed using a multivariate logistic regression analysis with an extended bootstrapping technique as described by El Naqa et al. [7] and Beetz et al. [3]–[5]. For each patient, predictive values were calculated for each set of prognostic factors based on the multivariate logistic regression coefficients according to the formula:
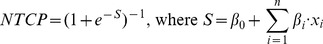
(1)in which *n* is the number of prognostic factors in the built model; variables *x_i_* represent different prognostic factors; and *βi* are the corresponding regression coefficients.

For each patient, 16 candidate prognostic factors were initially included in the variable selection procedure. The candidates included 14 clinical and two dosimetric factors. The clinical candidate factors were chemotherapy (C/T), treatment mode (SIB or SQM), gender, age, AJCC stage, baseline xerostomia, T stage, node classification, education, family history, financial status, marriage, smoking, and alcohol abuse.

The dosimetric candidate factors were the mean dose given to the contralateral parotid gland (Dmean-c) and the ipsilateral parotid gland (Dmean-i) (Gy). We excluded Vx values, which were previously found to be highly correlated with each other [4]; Dmean-c and Dmean-i were the only two DVH-parameters in this study. The candidate prognostic factors are listed in Table 2. We used the LASSO process along with the Hosmer-Lemeshow test to select the optimal and suboptimal numbers of potential prognostic factors for the predictive model.

**Table 2:** Candidate prognostic factors initially in the xerostomia dataset.

No.	Description	Range or Classification	(3m) Median or frequency	(3m) correlation	(12M) Median or frequency	(12m) correlation
1	Dmean-c	4.9–68.3(Gy)	30.6	0.110	31.4	0.207
2	Dmean-i	12.2–70(Gy)	36.3	0.109	36.6	0.188
3	Age	26–89	51.7	0.283	50.4	0.082
4	Gender	0, 1#	25, 160	−0.215	17, 100	0.479
5	Education	0, 1, 2, 3	11, 32, 110, 32	$I	5, 12, 74, 26	$V
6	Marriage	0, 1*	32, 153	0.196	20, 97	0.526
7	Smoking	0, 1*	60, 125	−0.389	45, 72	0.870
8	Alcohol abuse	0, 1*	73, 112	0.256	51, 66	0.941
9	AJCC stage	1, 2, 3, 4	22, 18, 38, 107	$II	9, 16, 27, 65	$VI
10	T stage	1, 2, 3, 4	41, 64, 15, 65	$III	31, 37, 9, 40	$VII
11	Node classification	0, 1	19, 166	0.785	12, 105	1.070
12	Chemotherapy (C/T)	0, 1*	33, 152	0.514	20, 97	−0.696
13	Baseline xerostomia	0, 1	75, 110	0.397	54, 63	0.851
14	Family history	0, 1*	134, 51	0.180	83, 34	−0.633
15	Financial status	0, 1, 2, 3	63, 94, 21, 7	$IV	40, 56, 17, 4	$VIII
16	SIB or SQM	0, 1	88, 93	0.274	66, 48	0.033

*Abbreviation*: AJCC: American Joint Committee on Cancer; SIB: simultaneous integrated boost; SQM: sequential mode;

Dmean-c: mean dose to the contralateral parotid glands; Dmean-i: mean dose to the ipsilateral parotid glands;

# 0 = Female, 1 = Male;

* 0 = No, 1 = Yes;

Education: 0 = elementary, 1 = junior, 2 = senior, 3 = university;

AJCC stage: 1 = stage 1, 2 = stage 2, 3 = stage 3, 4 = stage 4;

T stage: 0 = T0, 1 = T1, 2 = T2 (T2a, T2b), 3 = T3, 4 = T4;

Node classification: 0 = N0, 1 = N1, 2 = N2, 3 = N3 (N3a, N3b);

Baseline xerostomia: xerostomia before RT, 0 = No, 1 = a little;

Financial: 0 = under 16.7(f0), 1 = 16.7–33.3(f1), 2 = 33.3–66.7(f2), 3 = more than 66.7(f3); (unit: Thousand dollars);

$I: E(0) = 0, E(1) = 0.032, E(2) = 0.541, E(3) = 0.213;

$II: stage 1 = 0, stage 2 = −0.684, stage 3 = 0.091, stage 4 = 0.527;

$III: T(1) = 0, T(2) = −0.524, T(3) = −1.277, T(4) = −0.315;

$IV: f(0) = 1, f(1) = −0.431, f(2) = 0.842, f(3) = −0.365;

$V: E(0) = 0, E(1) = −3.947, E(2) = −0.938, E(3) = −0.795;

$VI: stage 1 = 0, stage 2 = −0.179, stage 3 = −0.871, stage 4 = −0.388;

$VII: T(1) = 0, T(2) = 0.071, T(1) = −0.839, T(2) = 0.91;

$VIII: f(0) = 0, f(1) = 0.445, f(2) = 0.175, f(3) = 1.075;

The LASSO was first proposed by Tibshirani in 1996, the details of which can be found in [22]. It uses the following equation to shrink the coefficients and select the prognostic factors:
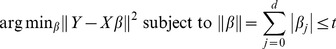
(2)where *d* is the number of variables selected, and *t* are tuning parameters that control the degree of penalty, which can be determined by cross-validation [9], [23]. Based on the nature of the constraint, LASSO tends to produce some coefficients as zero, and it improves the overall prediction accuracy by allowing a small amount of bias to reduce the variance of the predicted values. The selection of the optimal number of prognostic factors was performed by cross-validation in this study.

To account for the overfitting problem in our study, two datasets were used, i.e., a training set and a test set; a model was built based on a training set and fitted to the training set itself and also tested with a test set. We used nested 10-fold cross-validation to obtain the best prognostic factor subsets.

To generalize the use of the models, a compact model can be generated with a small suboptimal number of prognostic factors for more user-friendly handling; however, accuracy has to remain at a certain level. For the selection of a suboptimal number of prognostic factors, we first processed the LASSO to rank the correlations for the potential prognostic factors, i.e., the DVH parameters and patients' clinical data. In order to reduce the number of prognostic factors in the model, the suboptimal number of prognostic factors for a multivariate logistic regression model was determined using a bootstrapping method with the Hosmer-Lemeshow test. We set the Hosmer-Lemeshow test to show a significant agreement between predicted risk and observed outcome when the Hosmer-Lemeshow test value was ≥0.05. The first number needed for the model was recorded when the value was ≥0.05, after which we put the selected prognostic factors into the model to calculate the area under the receiver operating characteristic curve (AUC). We carried on increasing the number of prognostic factors with a higher Hosmer-Lemeshow test value and stopped when the system AUC did not increase significantly (<5%). The Hosmer-Lemeshow test revealed the accuracy of the model. The AUC was also used as a measure to evaluate prediction performance and it described the discriminatory ability of the model. The prognostic factors selected were used for the definitive NTCP model for moderate-to-severe patient-rated xerostomia.

After selecting the prognostic factors with optimal or suboptimal performance, odds ratios (OR) and 95% confidence intervals (95% CI) were calculated for these factors. Model performance was described using different validation tools [3]–[6]. The system's performance was evaluated using the AUC, scaled Brier score, Nagelkerke R^2^, Omnibus, and Hosmer-Lemeshow test [3]–[6]. Statistical analyses were performed using SPSS 19.0 (SPSS, Chicago, IL, USA).

### Forward selection

Beetz et al. used the forward selection method to develop NTCP models for moderate-to-severe patient-rated xerostomia using a multivariate logistic regression analysis with an extended bootstrapping technique and forward variable selection [3], [4]. We compared their method with the LASSO mentioned above. For every model order, the likelihood of predictions was calculated and the number of selected variables with the highest likelihood was selected for the definitive predictive model for patient-rated xerostomia. Two thousand bootstraps were used for each analysis. The details can be found in [3], [4].

## Results

All patients completed QoL questionnaires at three time points (before RT, during RT, and at 3 months after RT). In addition, 128 patients completed a QoL questionnaire 12 months after IMRT. Of the 206 patients assessed at the 3-month time point, 21 who were already suffering from moderate-to-severe xerostomia at baseline were excluded, leaving 185 patients for analysis. Of the 128 patients assessed at the 12-month time point, 11 who were already suffering from moderate-to-severe xerostomia at baseline were excluded from further analysis, leaving 117 patients for analysis.

At 3 months after treatment, 42.2% of the patients reported moderate- to-severe xerostomia. After 12 months, 33.6% reported moderate-to-severe xerostomia (Table 1).

**Figure 1:**
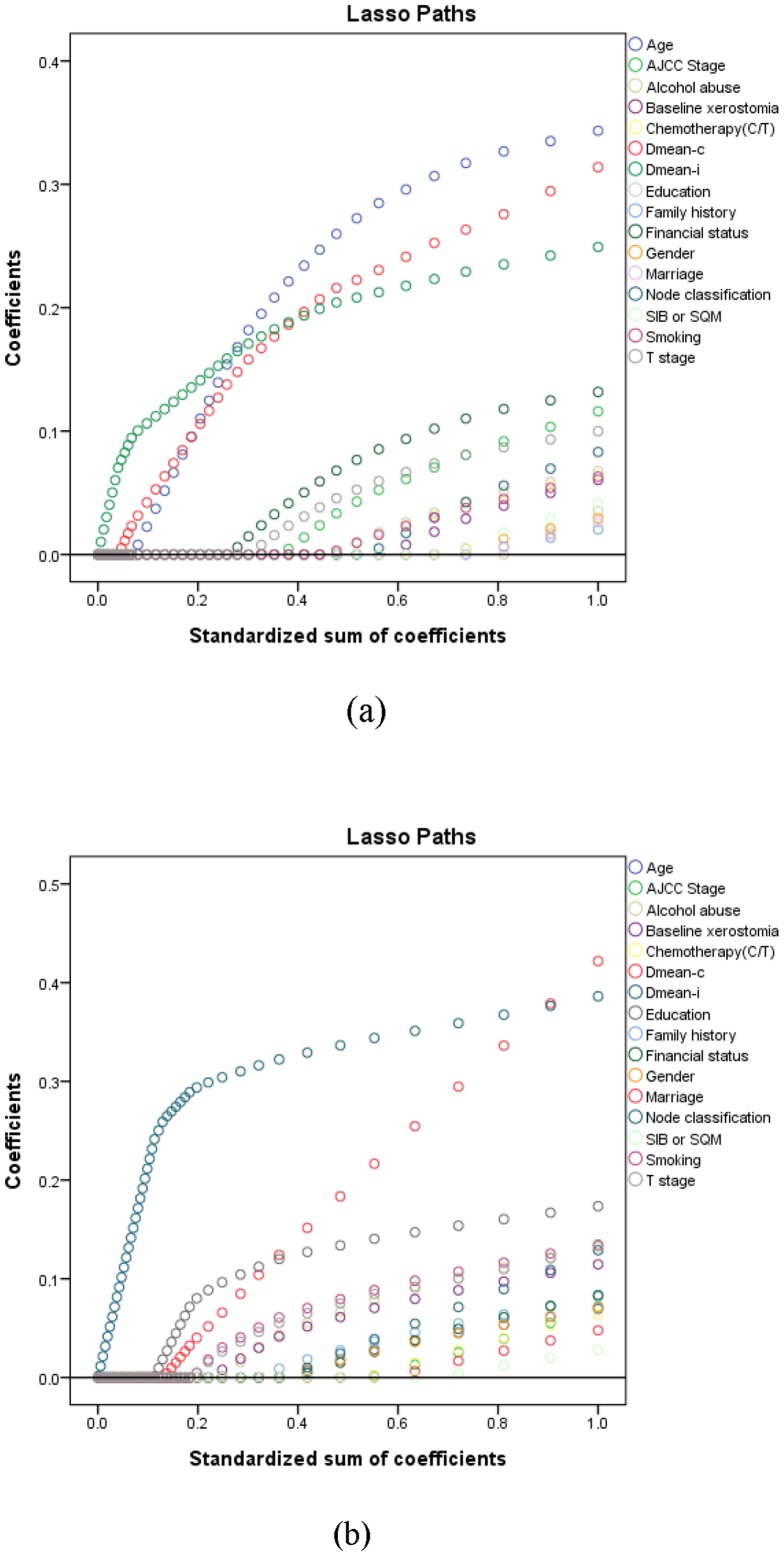
The LASSO shrinking path diagrams at (a) 3- and (b) 12-month time point. *Abbreviation*- LASSO: least absolute shrinkage and selection operator; Definition of prognostic factors: Same as Table 2.

The following steps were used to select the optimal and suboptimal numbers of prognostic factors. First, LASSO ranked how strongly the factors correlated, then chose the suboptimal number of prognostic factors using the Hosmer-Lemeshow test and AUC. LASSO of bootstrap prediction in the multivariate logistic regression analysis ranked the prognostic factors in descending order, as shown in Table 3 (the LASSO shrinking path diagrams are shown in Figs. 1a and b) for both the 3- and 12-month time points.

**Table 3:** Prognostic factors correlation ranking for the 3- and 12-month time points by LASSO.

**XER_3m_**			
1.Dmean-c	6.AJCC Stage	11. Baseline xerostomia	16. Alcohol abuse
2.Dmean-i	7.Smoking	12. SIB or SQM	
3.Age	8.Education	13. Gender	
4.Financial status	9.Chemotherapy(C/T)	14. Family history	
5.T stage	10.Node classification	15. Marriage	
**XER_12m_**			
1.Dmean-i	6. Baseline xerostomia	11.Age	16. SIB or SQM
2.Education	7. Alcohol abuse	12. Financial status	
3.Dmean-c	8. Family history	13. Chemotherapy(C/T)	
4. Smoking	9. Node classification	14. AJCC Stage	
5.T stage	10. Gender	15. Marriage	

*Abbreviation*: AJCC: American Joint Committee on Cancer; XER_3m_: xerostomia at 3-month time point; XER_12m_: xerostomia at 12-month time point; SIB: simultaneous integrated boost; SQM: sequential mode; Dmean-c: mean dose to the contralateral parotid glands; Dmean-i: mean dose to the ipsilateral parotid glands;

Eight prognostic factors were selected by LASSO for the 3-month time point: Dmean-c, Dmean-i, age, financial status, T stage, AJCC stage, smoking, and education. For the 12-month time point, nine prognostic factors were selected: Dmean-i, education, Dmean-c, smoking, T stage, baseline xerostomia (none vs. a little), alcohol abuse, family history, and node classification.

For the 3-month time point, three suboptimal prognostic factors were selected by the Hosmer-Lemeshow test and AUC. We set the Hosmer-Lemeshow test to show a significant agreement between predicted risk and observed outcome when the Hosmer-Lemeshow test value was ≥0.05. Increasing the number of variables to four or eight did not further increase the AUC of the model compared with the three-factor model, i.e., Dmean-c, Dmean-i, and age.

For the 12-month time point, five suboptimal prognostic factors were selected by the Hosmer-Lemeshow test and AUC, i.e., Dmean-i, education, Dmean-c, smoking and T stage. Increasing the number of variables to five or seven did not further increase the AUC of the model compared with the five-factor model.

All corresponding coefficients of the multivariate logistic regression NTCP models are shown in Tables 4 and 5. The NTCP value for each individual patient can be calculated using the following logistic regression formula:

**Table 4:** Multivariate logistic regression coefficients and odds ratios for the NTCP models for patient-rated xerostomia 3 and 12 months after treatment for the optimal prognostic factors selection.

	prognostic factors	β	*p*	Odds Ratio	95% CI
XER_3m_	(n = 8)				
	Dmean-c	0.102	<0.001	1.108	1.078–1.139
	Dmean-i	0.102	<0.001	1.108	1.075–1.142
	Age	0.292	<0.001	1.339	1.277–1.404
	Financial status				
	f(0)	0	<0.001		
	f(1)	−0.452	0.005	0.636	0.464–0.872
	f(2)	0.904	<0.001	2.471	1.512–4.036
	f(3)	−0.086	0.833	0.918	0.413–2.039
	T stage				
	stage(1)	0.272	0.255	1.313	0.822–2.097
	stage(2)	−0.25	0.201	0.779	0.531–1.143
	stage(3)	−1.404	<0.001	0.246	0.118–0.509
	stage(4)	0	<0.001		
	AJCC stage				
	stage(1)	0	0.001		
	stage(2)	−0.481	0.176	0.618	0.308–1.24
	stage(3)	0.455	0.128	1.576	0.878–2.831
	stage(4)	0.560	0.033	1.751	1.046–2.931
	Smoking	0.187	0.255	0.830	0.602–1.144
	Education				
	E(1)	0	0.034		
	E(2)	−0.009	0.981	0.991	0.472–2.08
	E(3)	0.519	0.127	1.680	0.863–3.273
	E(4)	0.207	0.580	1.230	0.591–2.561
	constant	−22.283	<0.001		
XER_12m_	(n = 9)				
	Dmean-i	0.176	<0.001	1.193	1.149–1.28
	Education				
	E(1)	0	<0.001		
	E(2)	−4.097	<0.001	0.017	0.004–0.062
	E(3)	−1.346	0.005	0.260	0.101–0.671
	E(4)	−1.165	0.023	0.312	0.115–0.849
	Dmean-c	0.121	<0.001	1.129	1.094–1.165
	Smoking	0.993	<0.001	2.700	1.903–3.829
	T stage				
	T(1)	−0.963	<0.001	0.382	0.245–0.595
	T(2)	−1.048	<0.001	0.350	0.239–0.514
	T(3)	−1.522	<0.001	0.218	0.107–0.446
	T(4)	0	<0.001		
	Baseline xerostomia	0.898	<0.001	2.455	1.745–3.453
	Alcohol abuse	0.913	<0.001	2.492	1.743–3.562
	Family history	−0.871	<0.001	0.419	0.290–0.604
	Node classification	1.034	<0.001	2.812	1.822–4.339
	constant	−12.045	<0.001	0	

*Abbreviation*: AJCC: American Joint Committee on Cancer;

Definition of prognostic factors: Same as Table 2.

**Table 5:** Multivariate logistic regression coefficients and odds ratios for the NTCP models for patient-rated xerostomia 3 and 12 months after treatment for the suboptimal prognostic factors selection.

	prognostic factors	β	*p*	Odds Ratio	95% CI
XER_3m_	(n = 3)				
	Dmean-c	0.097	<0.001	1.101	1.076–1.128
	Dmean-i	0.101	<0.001	1.106	1.075–1.138
	Age	0.285	<0.001	1.329	1.272–1.390
	constant	−21.298	<0.001		
XER_12m_	(n = 5)				
	Dmean-i	0.155	<0.001	1.167	1.128–1.208
	Education				
	E(1)	0	<0.001		
	E(2)	−3.888	<0.001	0.020	0.006–0.701
	E(3)	−1.294	0.004	0.274	0.115–0.654
	E(4)	−1.242	0.008	0.289	0.115–0.725
	Dmean-c	0.067	<0.001	1.069	1.040–1.098
	Smoking	1.143	<0.001	3.138	2.256–4.364
	T stage				
	T(1)	−0.958	<0.001	0.384	0.253–0.582
	T(2)	−1.077	<0.001	0.341	0.236–0.491
	T(3)	−1.374	<0.001	0.253	0.131–0.488
	T(4)	0	<0.001		
	constant	−8.028	<0.001	0.001	

Definition of prognostic factors: Same as Table 2.

For the 3-month time point, the optimal model was where S = −22.283+(Dmean-c*0.102)+(Dmean-i*0.102)+(Age*0.292)+(financial status*corresponding coefficient)+(T stage*corresponding coefficient)+(AJCC stage*corresponding coefficient)+(smoking*0.187)+(education*corresponding coefficient). The suboptimal model was where S = −21.298+(Dmean-c*0.097)+(Dmean-i*0.101)+(Age*0.285).

For the 12-month time point, the NTCP value for each individual patient can be calculated by the above logistic regression formula, and where S = −12.045+(Dmean-i*0.176)+(education*corresponding coefficient)+(Dmean-c*0.121)+(smoking*0.993)+(T stage*corresponding coefficient)+(baseline xerostomia*0.898)+(alcohol abuse*0.913)+(family history*−0.871)+(node classification*1.034). The suboptimal model was where S = −8.028+(Dmean-i*0.155)+(education*corresponding coefficient)+(Dmean-c*0.067)+(smoking*1.143)+(T stage*corresponding coefficient).

**Figure 2:**
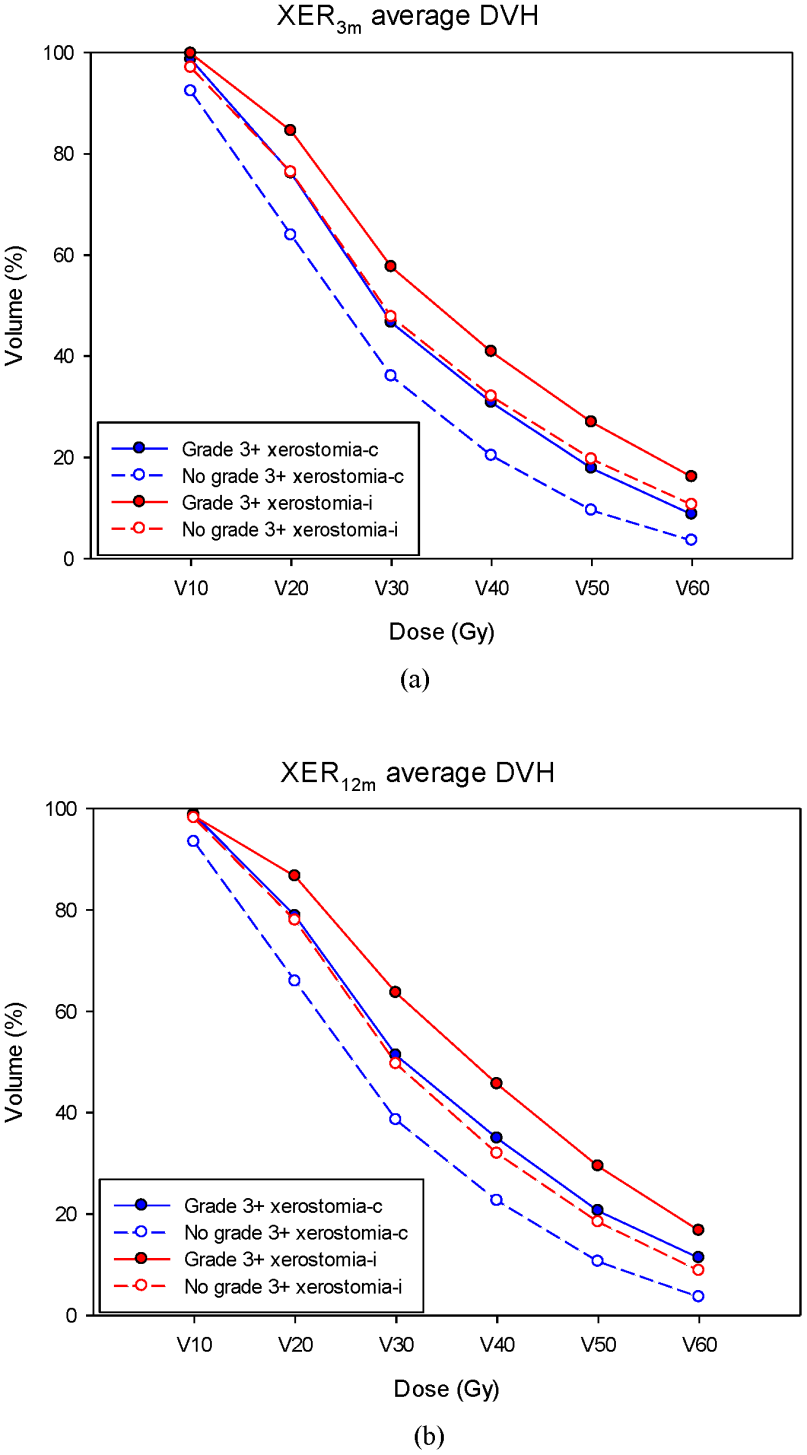
The average DVH diagrams for the mean doses delivered to the ipsilateral and contralateral parotids in the group with/without grade 3^+^ xerostomia at (a) 3- and (b) 12-month time point. Abbreviation- DVH: dose–volume histogram; XER_3m_: xerostomia at 3-month time point; XER_12m_: xerostomia at 12-month time point.

The overall performance for both time points of the NTCP model for patient-rated xerostomia in terms of scaled Brier score, Omnibus, and Nagelkerke R^2^ was satisfactory and corresponded well with the expected values (Table 6). The AUC for the suboptimal model was 0.84 (95% CI 0.80–0.86) and 0.84 (95% CI 0.81–0.86) for 3 and 12 months, respectively. The AUC for the optimal model was only slightly improved (less than 5%) compared with the suboptimal model; this was 0.86 (95% CI 0.83–0.88) and 0.87 (95% CI 0.84–0.89) for 3 and 12 months, respectively. Finally, the Hosmer-Lemeshow test showed significant agreement between predicted risk and observed outcome for both LASSO optimal and suboptimal models (Table 6). In our population, the DVH diagrams for the mean doses delivered to the ipsilateral and contralateral parotids are shown in Fig. 2 for both the 3- and 12-month time points.

**Table 6:** System performance evaluation.

	Number of factors	AUC	Scaled Brier-score	R^2^ Nagelkerke	Omnibus	Hosmer–Lemeshow
XER_3m_(lasso-suboptimal)	3	0.84 (0.80–0.86)	0.33	0.327	<0.001	0.327
XER_3m_(lasso-optimal)	8	0.86 (0.83–0.88)	0.36	0.455	<0.001	0.694
XER_3m_(LL)	9	0.85 (0.83–0.88)	0.38	0.461	<0.001	0.676
XER_12m_(lasso- suboptimal)	5	0.84 (0.81–0.86)	0.32	0.432	<0.001	0.060
XER_12m_(lasso- optimal)	9	0.87 (0.84–0.89)	0.39	0.511	<0.001	0.101
XER_12m_(LL)	11	0.86 (0.83–0.88)	0.35	0.525	<0.001	0.916

*Abbreviation*: AUC: Area under the receiver operating characteristic curve; LL: likelihood; XER_3m_: xerostomia at 3-month time point; XER_12m_: xerostomia at 12-month time point;

For the likelihood forward selection, the numbers of prognostic factors selected were 9 and 11 for the 3- and 12-month time points, respectively. The AUC of the forward selection model was similar to that achieved by LASSO: 0.85 (95% CI 0.83–0.88) and 0.86 (95% CI 0.83–0.88) for 3 and 12 months, respectively.

## Discussion

Early NTCP models, like the Lyman-Kutcher-Burman and the univariate logistic regression model [24], are based on information derived from DVHs generated from dose distributions in the target volumes and the surrounding organs at risk. For example, the mean dose received in the parotid glands is the only prognostic factor for xerostomia in univariate models. In this multivariate model study, not only the mean doses to the contralateral/ipsilateral parotid glands were the principal components causing xerostomia; but also age, education, and smoking were also to be the effected components.

For the IMRT planning goal, the mean dose to each parotid gland should be kept as low as possible, consistent with the desired clinical target volume coverage. Sparing at least one parotid gland appears to eliminate complications [2], [25]. Severe xerostomia can usually be avoided if at least one parotid gland has been given a mean dose ≤20 Gy or if both glands have been given a mean dose ≤25 Gy. A lower parotid mean dose usually results in better function with respect to the effects on patients' QoL [2], [6], [25].

In our population, the average mean doses to the ipsilateral and contralateral parotid were 36.3 Gy vs. 30.6 Gy, respectively. These doses were more or less similar to those observed in previous IMRT treatment reports, such as Beetz et al. [26], who found that they were 35.2 Gy and 28.0 Gy to the ipsilateral and contralateral parotid gland, respectively; Nutting et al. [27] reported that the average mean doses to the ipsilateral and contralateral parotid glands were 47.6 Gy and 25.4 Gy, respectively. In our cohort, these average dose levels were much higher in the subset of nasopharyngeal cancer patients: 41.6 Gy and 38.7 Gy to the ipsilateral and contralateral gland, respectively. A possible explanation for this is that the overlap of the PTV with parts of the parotid glands in nasopharyngeal cancer patients makes it more difficult to spare the parotid glands with IMRT [26]. This implies that more effective new technologies that could spare the parotid glands without compromising target volume coverage need to be investigated.

The current analysis, for the 3-month time point, showed that the mean dose to the contralateral parotid gland was the most important variable determining acute xerostomia. The three-factor model containing the mean dose to the contralateral gland and the ipsilateral gland performed significantly better than the single variable model. The dose to the contralateral parotid gland was more important than the dose to the ipsilateral parotid gland when treated with IMRT; similar reports can be found in a number of papers on patient-rated xerostomia [4], [26], [28]–[30].

For the 12-month time point, the dose to the contralateral parotid gland was also significantly correlated with late xerostomia. However, the mean dose to the ipsilateral parotid gland was the most important variable. As the mean dose to the contralateral parotid gland was significantly lower in patients with/without xerostomia when treated with IMRT, it is not surprising that recovery can be expected during this period. Indeed, some investigators showed that the contralateral parotid recovered after 9 to 24 months in different recovery levels. Eisbruch et al. [29] reported the correlation between mean parotid dose and parotid salivary flow recovery in patients with non-nasopharyngeal head-and-neck cancer. In the Kwong et al. [31] study, 60% of patients recovered at least 25% of their baseline stimulated parotid salivary flow at 12 months post-IMRT. In Hsiung et al. [32], 64.5% of 31 parotid glands recovered at least 25% of their baseline maximal excretion ratio at 9 months post-IMRT. This was consistent with the results of another study [33], which suggested that reducing radiotherapy doses to both parotid glands to <26 Gy can reduce xerostomia significantly.

Although parotid gland dysfunction plays an important role in the development of patient-rated xerostomia [3], it is not the only prognostic factor. Beetz et al. recently showed that age and baseline xerostomia were independent prognostic factors for patient-rated xerostomia, in addition to the mean dose to the parotid glands [3], [26]. In the current study, we likewise found that elderly patients have a higher probability of suffering from xerostomia than younger patients. Beetz et al. claimed that older patients are more likely to use medication and to have co-morbidities that may influence and reduce saliva production at rest [3], [34]. Fortin et al. [35] showed that in a univariate and multivariate analysis, total parotid mean dose and age were strongly associated with a lower incidence of grade ≥2 xerostomia at 6, 12 and 24 months.

In this study, those who had a higher financial status or a higher level of education tended to avoid the inconvenience of xerostomia. Similarly, Ramsey et al. [26] showed that lower financial status in colorectal cancer patients was associated with a worse outcome for reported pain. Fang et al. [21] found that NPC survivors with a higher annual family income and level of education presented a significantly better outcome on QoL scores. These findings suggest that the patient's individual abilities and the resources available to cope with the threat of treatment complications are powerful variables that affect their future quality of life. Financial status and education remain two of the most significant variables correlated with xerostomia in our cohort. In addition, smoking, a recognized carcinogenic factor in the overwhelming majority for head and neck cancers [36], [37], was shown to be a contributory cause of xerostomia after IMRT in our cohort. Rad et al. [38] also indicated that long-term smoking would significantly reduce whole-mouth salivary flow rate.

That the risk of complications may depend on more factors than simply the dose to a single organ seems to be true. Clinical datasets on normal tissue complications often include a large number of variables, many of which need to be investigated and incorporated in a model because they may be related to a given complication. As reported by El Naqa et al., the prediction of endpoints can be improved by mixing clinical and dose-volume factors, while bootstrap-based variable selection analysis increases the reliability of the predictive models [7]. Indeed, our results showed better performance of the multivariate model compared with the univariate relationships between dose–volume prognostic factors and XER_3m_or XER_12m_.

Recently, we reported the results of a retrospective study that was conducted to develop a univariate NTCP model for patient-rated moderate-to-severe xerostomia among HNC patients treated with IMRT [5]. The AUC values for the model were 0.68 (95% CI 0.61–0.74) and 0.72 (95% CI 0.64–0.80) for the 3- and 12-month time points, respectively. The only prognostic factor was the mean dose to parotid glands. In this study, for the selection of suboptimal prognostic factors, the system performance AUC values improved from 0.68 to 0.84 for the 3-month time point, and from 0.72 to 0.84 for the 12-month time point when the number of prognostic factors used increased from one to three or four, respectively. The multivariate approach allowed the integration of different prognostic factors for estimating the risk on XER_3m_ and XER_12m_ in individual patients. In this regard, it should be emphasized that dose–effect relationships for this endpoint should be described by multiple NTCP curves rather than by a single NTCP curve. Moreover, whether the gain is worth the increased complexity requires further investigation. The problem of increased complexity is the potential limitation of this study. After all, a large number of selected prognostic factors may lead to instability in the models.

In the literature, a similar report by Xu et al. stated that the NTCP model obtained by LASSO was statistically significant [10]. Their dataset contained 185 patients who were all treated with primary 3D-CRT for HNC at 6 months after RT; 106 patients were assessed as having xerostomia. The primary endpoint was defined as Radiation Therapy Oncology Group (RTOG) grade 2^+^ xerostomia using the RTOG late radiation morbidity scoring system. The prognostic factors used for the NTCP modeling included five clinical and 16 dosimetric factors. The five clinical variables were chemotherapy, gender, age, treatment center, and baseline xerostomia score. The dosimetric factors were the mean dose given to the organs at risk and their volume. These organs were the lower lip, the soft palate, the contralateral and ipsilateral parotid gland, the contralateral and ipsilateral sublingual gland, and the contralateral and ipsilateral submandibular gland. The differences in comparison with our models included treatment technique (IMRT vs. 3D-CRT), time point (3- and 12-month vs. 6-month), endpoint (G3^+^ vs. G2^+^), and candidate prognostic factors (14 clinical and two dosimetric factors vs. five clinical and 16 dosimetric factors). However, the system performance AUC values were similar with both regression models (approximately 0.85). Therefore, LASSO was recommended for multivariate logistic regression NTCP modeling.

The LL forward selection of bootstrap predictions in the multivariate logistic regression analysis was optimal with a model consisting of 9 and 11 variables for the 3- and 12-month time points, respectively. The system performance AUC values were no better than the results of LASSO. However, the selection of too many prognostic factors may result in overfitting.

There are a number of potential limitations of this study. Arjen van der Schaaf et al. [39] claimed that approximately 200 patients are required to obtain a model with high predictive power. In the current study, the number of patients evaluable at 12 months was far below the recommended 200 patients. The predictive power can be improved by increasing the sample size. Data from 6 months were not evaluated on the basis of previously published data [40]–[42], in which the dose dependency of salivary function and recovery with time after RT was found up to 12 months post-RT. In addition, in the Kwong et al. [31] study, 60% of patients recovered at least 25% of their baseline stimulated parotid salivary flow at 12 months post-IMRT. Eisbruch et al. [28], [29] showed that a parotid mean dose <26 Gy decreases the severity of late complications and gives a better chance of functional parotid recovery at 12 months. The stability for evaluating late toxicity was more reliable at 12 months than 6 months.

That chemotherapy, a non-dosimetric patient factor, may affect the risk of xerostomia is an issue of special concern. Deasy et al. [25] and Moiseenko et al. [2] stated that the use of chemotherapy was not typically correlated with xerostomia risk. This is consistent with our results, as chemotherapy was not significant among the 16 candidate prognostic factors and there was no association between chemotherapy and risk of xerostomia.

## Conclusion

LASSO is recommended for multivariate NTCP modeling. However, in practice, the suboptimal LASSO method tends to incorporate fewer prognostic factors than the optimal approach. The suboptimal LASSO method is useful in practice. The prognostic factors included in the model are useful to further optimize current IMRT treatment with regard to patient-rated xerostomia and to indicate which prognostic factors are the most important, thus helping to spare the glands as much as possible, and to optimize current treatment with IMRT.
